# Dissimilar Effect of P-Glycoprotein and Breast Cancer Resistance Protein Inhibition on the Distribution of Erlotinib to the Retina and Brain in Humans and Mice

**DOI:** 10.1021/acs.molpharmaceut.3c00715

**Published:** 2023-10-26

**Authors:** Myriam El Biali, Sylvain Auvity, Salvatore Cisternino, Maria Smirnova, Marcus Hacker, Markus Zeitlinger, Severin Mairinger, Nicolas Tournier, Martin Bauer, Oliver Langer

**Affiliations:** † Department of Clinical Pharmacology, Medical University of Vienna, 1090 Vienna, Austria; ‡ Inserm UMRS1144, Optimisation Thérapeutique en Neuropsychopharmacologie, Université Paris Cité, F-75006 Paris, France; § Service Pharmacie, Assistance Publique-Hôpitaux de Paris, Hôpital Universitaire-Necker-Enfants Malades, F-75015 Paris, France; ∥ Division of Nuclear Medicine, Department of Biomedical Imaging and Image-guided Therapy, Medical University of Vienna, 1090 Vienna, Austria; ⊥ Laboratoire d’Imagerie Biomédicale Multimodale (BioMaps), CEA, CNRS, Inserm, Service Hospitalier Frédéric Joliot, Université Paris-Saclay, 91401 Orsay, France

**Keywords:** P-gp, BCRP, blood-retina barrier, blood-brain barrier, PET, in situ carotid perfusion, humans, mice, erlotinib, tariquidar

## Abstract

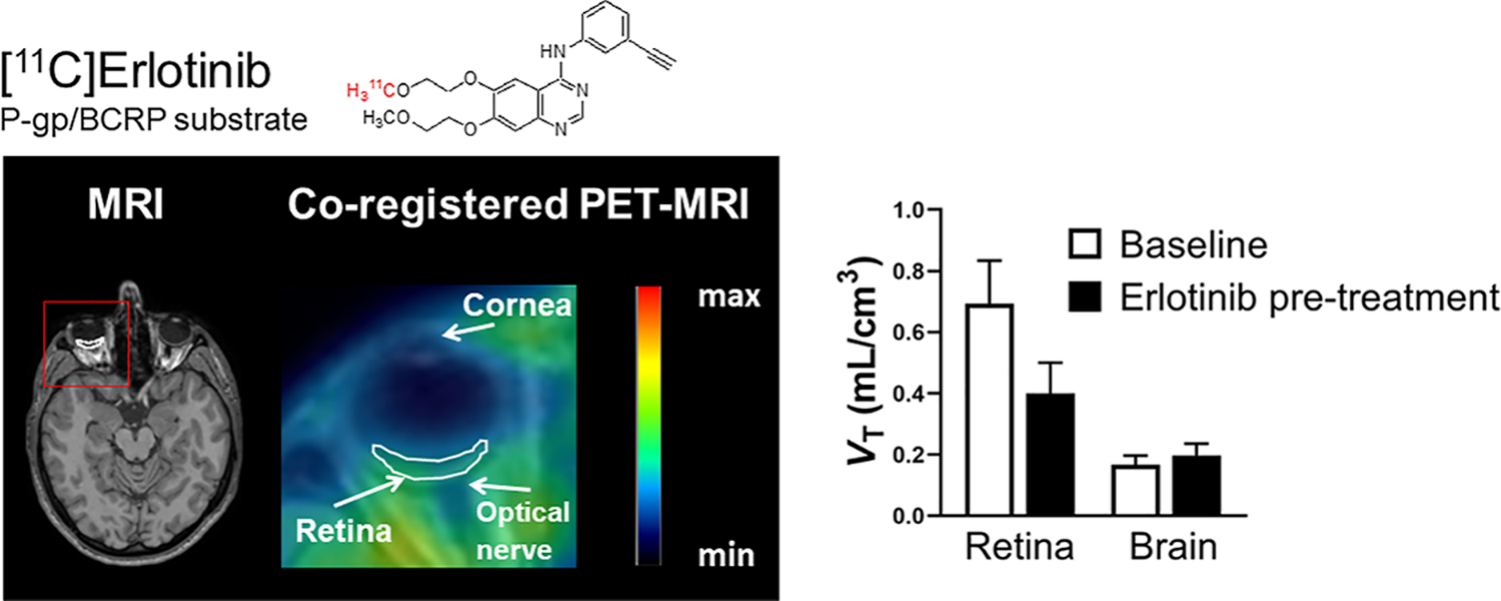

P-glycoprotein (P-gp) and breast cancer resistance protein (BCRP) are two ATP-binding cassette efflux transporters that are coexpressed at the human blood–brain barrier (BBB) and blood–retina barrier (BRB). While pharmacological inhibition of P-gp and/or BCRP results in increased brain distribution of dual P-gp/BCRP substrate drugs, such as the tyrosine kinase inhibitor erlotinib, the effect of P-gp and/or BCRP inhibition on the retinal distribution of such drugs has hardly been investigated. In this study, we used positron emission tomography (PET) imaging to assess the effect of transporter inhibition on the distribution of [^11^C]erlotinib to the human retina and brain. Twenty two healthy volunteers underwent two PET scans after intravenous (i.v.) injection of a microdose (<5 μg) of [^11^C]erlotinib, a baseline scan, and a second scan either with concurrent i.v. infusion of tariquidar to inhibit P-gp (*n* = 5) or after oral intake of single ascending doses of erlotinib (300 mg, 650 mg, or 1000 mg, *n* = 17) to saturate erlotinib transport. In addition, transport of [^3^H]erlotinib to the retina and brain was assessed in mice by in situ carotid perfusion under various drug transporter inhibition settings. In comparison to the baseline PET scan, coadministration of tariquidar or erlotinib led to a significant decrease of [^11^C]erlotinib total volume of distribution (*V*_T_) in the human retina by −25 ± 8% (*p* ≤ 0.05) and −41 ± 16% (*p* ≤ 0.001), respectively. In contrast, erlotinib intake led to a significant increase in [^11^C]erlotinib *V*_T_ in the human brain (+20 ± 16%, *p* ≤ 0.001), while administration of tariquidar did not result in any significant changes. In situ carotid perfusion experiments showed that both P-gp and BCRP significantly limit the distribution of erlotinib to the mouse retina and brain but revealed a similar discordant effect at the mouse BRB and BBB following co-perfusion with tariquidar and erlotinib as in humans. Co-perfusion with prototypical inhibitors of solute carrier transporters did not reveal a significant contribution of organic cation transporters (e.g., OCTs and OCTNs) and organic anion-transporting polypeptides (e.g., OATP2B1) to the retinal and cerebral distribution of erlotinib. In conclusion, we observed a dissimilar effect after P-gp and/or BCRP inhibition on the retinal and cerebral distribution of [^11^C]erlotinib. The exact mechanism for this discrepancy remains unclear but may be related to the function of an unidentified erlotinib uptake carrier sensitive to tariquidar inhibition at the BRB. Our study highlights the great potential of PET to study drug distribution to the human retina and to assess the functional impact of membrane transporters on ocular drug distribution.

## Introduction

Over the past 20 years, considerable efforts to increase the efficacy and reduce the systemic toxicity of conventional chemotherapy have led to the development of molecularly targeted anticancer drugs such as tyrosine kinase inhibitors. However, many of these compounds still exhibit various organ toxicities, including ocular and particularly retinal toxicity.^1,2^ Owing to its neuronal content, the retina is, like brain parenchyma, particularly vulnerable to toxic compounds and requires the strict maintenance of its homeostasis.^3^

The distribution of systemically administered drugs from blood to the retina is tightly regulated by the blood–retina barrier (BRB), which comprises the inner (iBRB) and the outer BRB (oBRB).^4,5^ The iBRB is formed by the endothelial cells from the intraretinal capillaries surrounded by pericytes, astrocytes, and Müller cells and is structurally similar to the blood–brain barrier (BBB), which is formed by the tight-junctional linking of brain capillary endothelial cells surrounded by astrocytes and pericytes. The oBRB is formed by the tight retinal pigment epithelium (RPE), which separates the neural tissue at its apical side and the blood (i.e., choroidal circulation) at its basolateral side.^4,5^

Efflux transporters of the ATP-binding cassette (ABC) family, i.e., P-glycoprotein (P-gp, encoded in humans by the *ABCB1* gene and in rodents by the *Abcb1a/b* genes) and breast cancer resistance protein (BCRP, encoded in humans by the *ABCG2* gene and in rodents by the *Abcg2* gene), are coexpressed at the luminal (blood-facing) membrane of brain capillary endothelial cells and are key determinants of the barrier function of the BBB.^6^ Dual substrates of P-gp and BCRP in most cases display a low brain distribution, as they are hindered by the combined action of P-gp and BCRP from crossing the BBB to reach the brain parenchyma. For significant penetration into cerebral tissues, impairment of the activity of both transporters is required. This phenomenon, called functional redundancy, has first been revealed in vivo at the rodent BBB^7,8^ and has also been confirmed in vivo at the human BBB.^9^

For the retina, evidence from in vitro and preclinical studies indicates that the iBRB expresses both P-gp and BCRP.^10−13^ However, at the oBRB, multidrug resistance-associated proteins (MRPs) are the most abundant ABC transporters, and P-gp and BCRP may either be absent or expressed only to a lower extent.^14−16^ However, the precise subcellular and membrane localization of ABC transporters and also of solute carrier (SLC) transporters at the BRB still needs further investigation.

The nuclear medicine imaging method positron emission tomography (PET) enables a noninvasive in vivo assessment of the distribution of radiolabeled drugs to the human eye. PET with radiolabeled model transporter substrates has been used to assess the activity of P-gp and BCRP at the human BRB.^17,18^ We successfully demonstrated a functional redundancy between P-gp and BCRP, comparable to that observed at the BBB, for the dual substrate [^11^C]tariquidar in humans at the overall BRB level.^17^

Erlotinib is a first-generation reversible and selective inhibitor of tyrosine kinase of the epidermal growth factor receptor (EGFR). It is approved for the treatment of locally advanced or metastatic EGFR mutation positive nonsmall cell lung cancer and pancreatic cancer. Its use has been linked to ocular toxicity, suggesting notable distribution to the eye in the case of systemic administration.^19^ In vitro as well as preclinical and human in vivo data indicate that erlotinib is transported by P-gp and BCRP^7,20−22^ and can also inhibit the activity of these two transporters.^23,24^ Single oral doses of 650 mg erlotinib were shown to enhance the brain distribution of a microdose of [^11^C]erlotinib, suggesting partial inhibition/saturation of its own transport by BCRP and P-gp at the BBB level.^22^ However, whether and to what extent P-gp and BCRP are involved in the retinal distribution of erlotinib is unknown.

In order to assess the influence of P-gp and BCRP on the distribution of [^11^C]erlotinib to the human retina, we extended the analysis of data from our previously published PET study in healthy volunteers that assessed the brain distribution of [^11^C]erlotinib.^22^ To obtain further insights into specific transport processes of erlotinib at the BRB and the BBB, we assessed [^3^H]erlotinib retina and brain distribution in mice in the presence and absence of selected SLC/ABC transporter inhibitors by in situ carotid perfusion.^11,25^

## Experimental Section

### Clinical PET Imaging

#### General

The PET data reported in this article derive from an extended analysis of a previously published study by Bauer et al.^22^ The study was registered with EUDRACT (number 2015-001593-18). It was approved by the Ethics Committee of the Medical University of Vienna and conducted in accordance with the Declaration of Helsinki and its amendments. Medication-free male (*n* = 20) and female (*n* = 2) subjects, who were judged to be healthy based on the screening examinations (past medical history, physical examination, blood and urine tests), were enrolled into the study and allocated to the tariquidar (*n* = 5) or erlotinib group (*n* = 17).

#### Imaging Procedure and Drug Administration Protocol

The volunteers (mean age: 31.9 ± 8.4 years) underwent two 60 min dynamic PET scans on an Advance scanner (GE Healthcare, Milwaukee, WI, USA), starting with an intravenous (i.v.) bolus injection of [^11^C]erlotinib over 20 s (injected radioactivity amount: 369 ± 22 MBq for the first scan and 372 ± 17 MBq for the second scan, containing less than 5 μg of unlabeled erlotinib). The second scan was performed on the same day for the tariquidar group or on a separate day for the erlotinib group. For the tariquidar group (*n* = 5), the second PET scan was performed with concurrent i.v. infusion of the P-gp inhibitor tariquidar (3.75 mg/min, AzaTrius Pharmaceuticals, Mumbai, India). The tariquidar infusion was started 1 h before the beginning of the PET acquisition and continued until the end of the PET acquisition with a total infusion length of 123 ± 4 min. The total administered dose of tariquidar was of 5.6 ± 0.6 mg/kg body weight. For the erlotinib group (*n* = 17), the second PET scan was acquired approximately 3 h after oral intake of 300 mg (*n* = 7), 650 mg (*n* = 8), or 1000 mg (*n* = 2) of erlotinib (Tarceva; Roche Pharma, 50 and 150 mg tablets).^22^ As described before, the occurrence of adverse events in the form of skin rash precluded the inclusion of additional subjects in the 1000 mg dose group.^22^ In addition to the PET scans, magnetic resonance imaging (MRI) of the head (T1-weighted; Magnetom Skyra 3.0-T MRI, Siemens Medical Solutions) was performed on all participants.

#### Blood and Metabolite Analysis

As described previously,^22^ serial arterial blood samples were collected during the two PET imaging sessions in both groups. Venous blood samples were collected at baseline and hourly for 8 h and at approximately 21 h after oral erlotinib intake for determination of erlotinib plasma concentrations. A gamma counter, which was cross-calibrated with the PET camera, was used to determine the radioactivity concentrations in blood and plasma aliquots. Due to the low percentage of radiolabeled metabolites of [^11^C]erlotinib in plasma (<10%), arterial plasma input functions were constructed without correction for radiolabeled metabolites.

#### PET and Pharmacokinetic Data Analysis

Volume of interest (VOI) analysis was conducted for the retina on MR-to-PET coregistered images using PMOD software (version 3.6; PMOD Technologies Ltd., Zürich, Switzerland). The retina VOI was outlined in a way to avoid spillover of radioactivity from adjacent anatomic structures (e.g., major blood vessels). The volume of the retina VOI was in the range of 0.8–0.9 cm^3^. None of the dimensions of the retina VOIs were much smaller than 2 times the full width at half-maximum (2 × 4.36 mm) of the employed PET scanner, meaning that partial volume effects causing an underestimation of the true radiotracer concentration in the VOI were negligible. Probabilistic atlas-based, whole brain gray matter (WBGM) data were already reported by Bauer et al.^22^ Time-activity curves (TACs) were extracted from the coregistered dynamic PET images and normalized to injected radioactivity amount per body weight and expressed in units of standardized uptake value (SUV). The area under the TACs in plasma (AUC_plasma_), the retina (AUC_retina_) and the brain (AUC_brain_), from 0 to 60 min after radiotracer injection, was determined with Prism 9.5.0 software (GraphPad Software, La Jolla, CA, USA). The ratio of AUC in tissue (retina or WBGM) to AUC_plasma_ (AUCR) was calculated as a parameter of the [^11^C]erlotinib distribution to the retina or brain, respectively. In addition, Logan graphical analysis was performed with the organ TACs and the arterial plasma input function using PMOD software to estimate the total volume of distribution (*V*_T_) in a model-independent manner for the retina and the brain.^26^*V*_T_ corresponds to the tissue-to-plasma concentration ratio of total radioactivity (i.e., comprising both bound and unbound [^11^C]erlotinib) at steady state.

### In Situ Carotid Perfusion

#### Animals and Chemicals

Male Swiss mice were purchased from Janvier (Le Genest Saint Isle, France). Animals (41.9 ± 0.9 g, 7–13 weeks) were housed in a controlled environment (22 ± 3 °C; 55 ± 10% relative humidity) with a 12 h dark/light cycle, with access to food and tap water ad libitum. The study was approved by the ethics review committee of Paris Descartes University (approval no. 12-183/12-2012). Study procedures complied with the ethical rules of the European directive (210/63/EU) for experimentation with laboratory animals. [^3^H]Erlotinib (molar activity: ≥33 GBq/mmol, radiochemical purity: ≥98%) was synthesized by Moravek Inc. (Brea, CA, USA) and purchased from Isobio sprl (Fleurus, Belgium). [^14^C]Sucrose (0.0037 MBq/mL) was purchased from PerkinElmer (Paris, France).

#### [^3^H]Erlotinib Transport Study

Transport of [^3^H]erlotinib at the luminal BRB and BBB was measured by in situ carotid perfusion.^11,25^ With this method, the vascular composition of the brain and the eye is completely substituted by an artificial fluid, whose constitution can be modified. The perfusion fluid for the in situ carotid perfusion consisted of Krebs carbonate-buffered physiological saline with 128 mM NaCl, 24 mM NaHCO_3_, 4.2 mM KCl, 2.4 mM NaH_2_PO_4_, 1.5 mM CaCl_2_, 0.9 mM MgSO_4_, and 9 mM d-glucose, warmed to 37 °C and gassed with 95% O_2_/5% CO_2_ to adjust the pH to 7.40. The pH of the perfusion fluid was checked and adjusted with a digital pH meter (±0.05 pH units) immediately before perfusion. Tissue accumulation of [^3^H]erlotinib was measured under trans-influx zero to determine the kinetic conditions required to measure transport solely across the membrane, separating the sucrose (vascular) from the nonsucrose (tissue parenchyma) space. The perfusion time adopted ensured that the tissue distribution of [^3^H]erlotinib was that of the initial linear part of the distribution kinetics. The first membrane (luminal/vascular) delimiting the sucrose space is the only kinetic interface that affects the distribution parameters of [^3^H]erlotinib.

Mice were anesthetized with ketamine and xylazine (140 and 8 mg/kg, intraperitoneal), and a catheter was inserted into the right carotid artery after ligation of the appropriate vessels. Just before perfusion, the heart was cut. Perfusion started immediately at a constant flow rate of 2.5 mL/min. Each mouse was perfused with [^3^H]erlotinib (0.011 MBq/mL, ∼ 0.33 μM) and [^14^C]sucrose as a vascular marker (0.003 MBq/mL) with or without the addition of selected transporter inhibitors [erlotinib, tariquidar, valspodar, elacridar, fexofenadine, tetraethylammonium (TEA), estrone-3-sulfate, ergothioneine, and l-carnitine]. Perfusion was terminated by decapitating the mouse after 60 s. The right eye (without the optic nerve) and the right cerebral hemisphere were removed from the skull and dissected on a freezer pack. The tissues and aliquots of perfusion fluid were weighted, digested (Solvable; PerkinElmer), and mixed with Ultima-gold XR (PerkinElmer). Dual-label counting was carried out in a Tri-Carb 2810TR instrument (PerkinElmer) to measure radioactivity (disintegrations per minute, dpm). Previous in situ carotid perfusion experiments had shown that, due to the short perfusion time, the small amount of radioactivity used, and the small size of the tissues, the distribution of radioactivity was limited to the richly irrigated posterior eye segment, and radioactivity in the vitreous and anterior segments was not quantifiable.^11,25^ Therefore, radioactivity in the whole eye was assumed to represent only the posterior segment of the eye, which comprises the retina.

#### Apparent Initial Tissue Distribution Volume and Transport Parameters

Calculations were performed as described previously.^11,25^ The initial transport rate, also called brain-eye clearance, expressed as *K*_in_ (μL/s/g) was selected as the main outcome parameter. The brain and eye tissue “vascular” volume (*V*_v_; μL/g) was estimated by using the [^14^C]sucrose distribution volume. The data for any mouse for which *V*_v_ was different from the normal values^25^ were excluded from the analysis.

### Statistical Analysis

All data are given as the arithmetic mean ± standard deviation (SD). Statistical analysis was performed with Prism version 9.5.0 software. The normal distribution of the values was assessed by visual inspection and the Shapiro–Wilk test. Differences in the PET imaging outcome parameters between scan 1 and 2 were tested using the two-sided paired *t*-test and between multiple groups using the mixed-effects analysis with the Geisser-Greenhouse correction and the Sidak’s multiple comparisons test. To assess correlations, the Pearson correlation coefficient *r* was calculated. Brain and eye *K*_in_ values of [^3^H]erlotinib in the presence of different SLC and ABC transporter inhibitors were compared to the control condition (no inhibitor) using one-way ANOVA with Dunnett’s multiple comparison test. The level of statistical significance was set to a *p*-value of less than 0.05.

## Results

### In Vivo Distribution of [^11^C]Erlotinib to the Retina and the Brain

**Figure 1:**
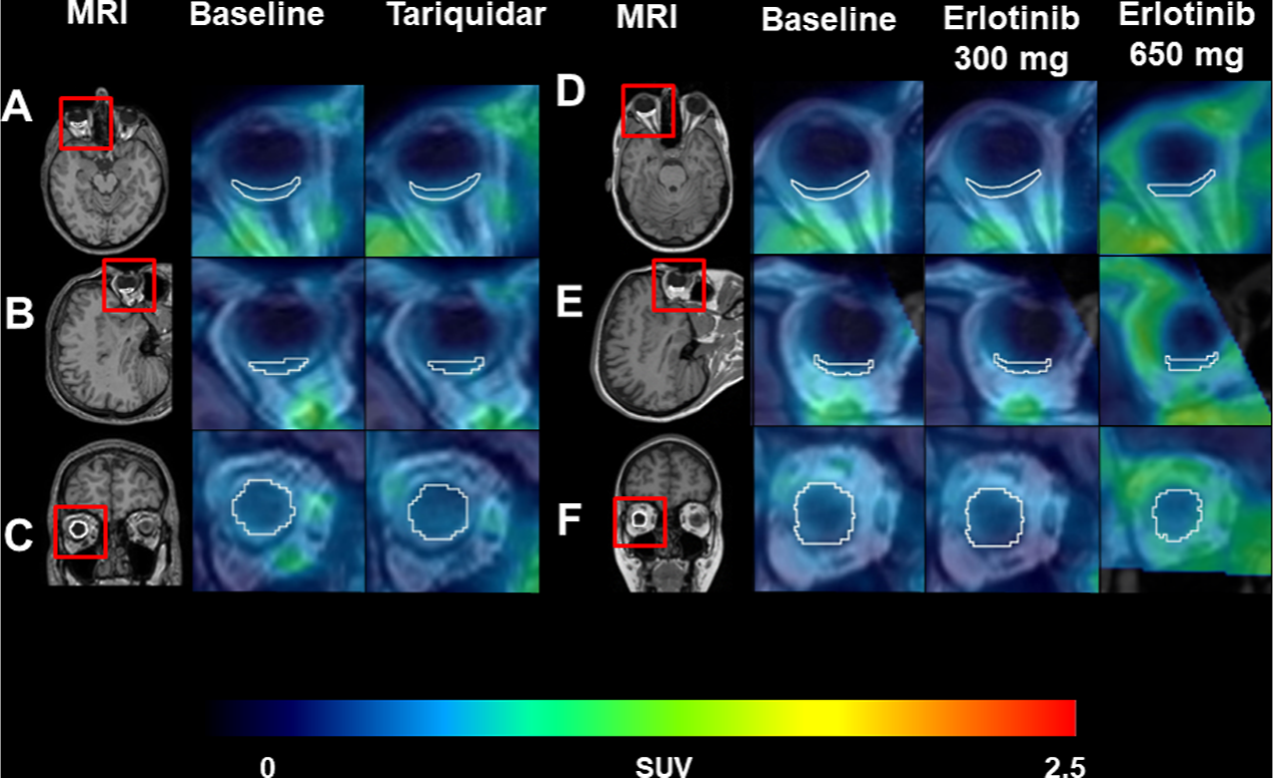
Axial (A,D), sagittal (B,E), and coronal (C,F) planes of representative MR and [^11^C]erlotinib PET average images (0–60 min) at baseline and after P-gp and/or BCRP inhibition with tariquidar (A–C) or with 300 or 650 mg erlotinib (D–F, MR image and baseline PET scan are only shown for the subject treated with 300 mg erlotinib). Red rectangles on MR images indicate magnified area on PET images. A representative region of interest for the retina (white contour) is shown. Radioactivity concentration is normalized to injected radioactivity amount per body weight and expressed as SUV. The radiation scale is set from 0 to 2.5.

To compare the influence of P-gp and BCRP at the BRB and the BBB on the retinal and cerebral distribution of erlotinib in humans, we performed two PET scans after i.v. injection of a microdose of [^11^C]erlotinib (<5 μg) in 22 healthy volunteers. The first PET scan was a baseline scan. The second PET scan was either performed concurrently with an i.v. infusion of tariquidar (*n* = 5) to inhibit P-gp or at 3 h after oral administration of single ascending doses (300, 650, or 1000 mg) of erlotinib (*n* = 17) to saturate erlotinib transport. Representative examples of the outlined retina VOI on MR and PET average images for the baseline scan and scans after the administration of tariquidar or erlotinib are shown in Figure 1.

**Figure 2:**
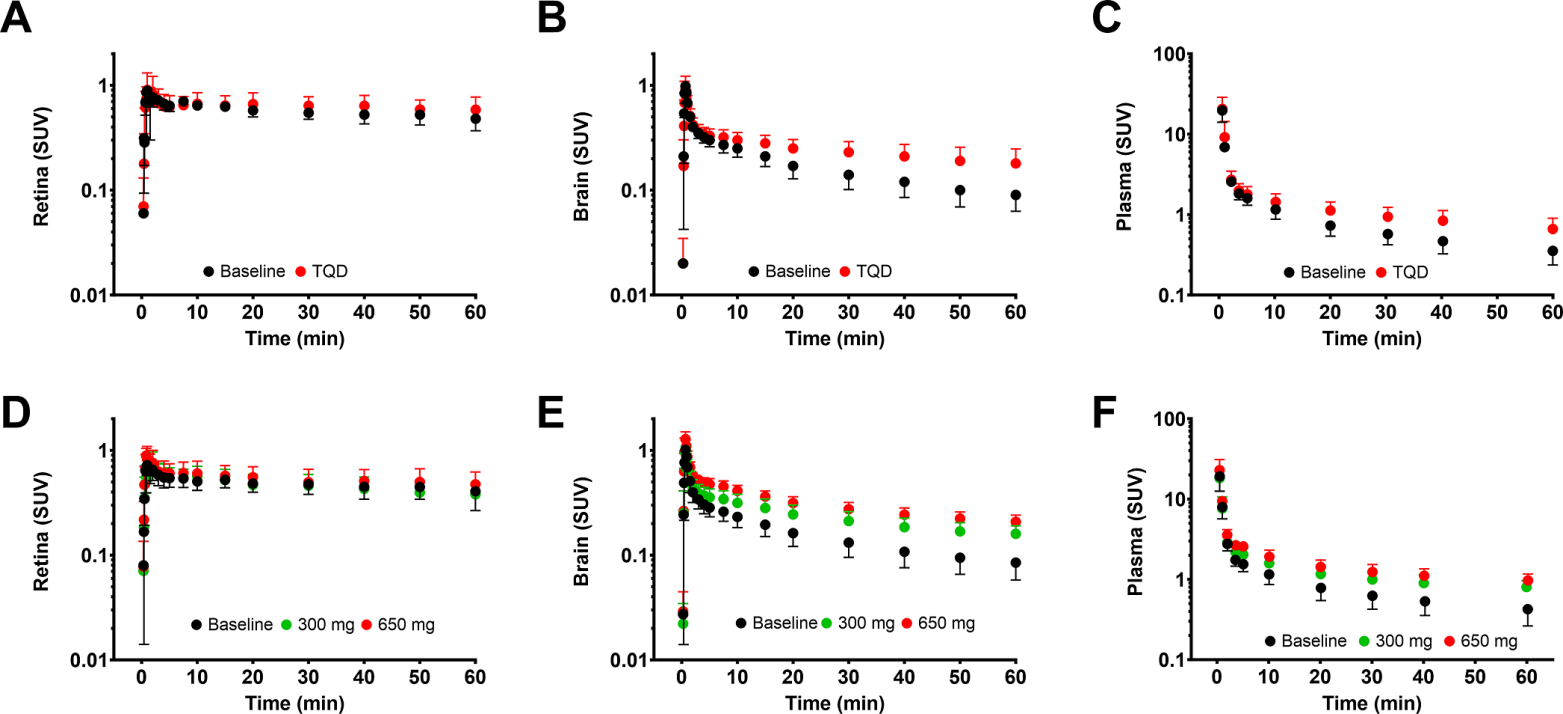
Mean time–activity curves (SUV ±SD) in the retina (A,D), whole-brain gray matter (B,E), and arterial plasma (C,F) for baseline scans (black circles) and scans during co-infusion of tariquidar (red circles) (A–C) or after oral intake of erlotinib at a dose of 300 mg (green circles) or 650 mg (red circles) (D–F).

TACs for baseline scans and scans with tariquidar infusion or oral erlotinib pretreatment are shown in Figure 2. Both treatments led to increases in the plasma and brain curves of [^11^C]erlotinib, while the retina curves remained unchanged (Figure 2).

Tissue distribution of [^11^C]erlotinib was expressed either as AUCR or as *V*_T_, calculated with Logan graphical analysis (Tables 1 and 2). The baseline [^11^C]erlotinib retinal distribution, expressed as *V*_T_, was 4 to 5 times higher than the baseline [^11^C]erlotinib brain distribution (0.89 ± 0.18 mL/cm^3^ versus 0.18 ± 0.02 mL/cm^3^ in the tariquidar group and 0.69 ± 0.14 mL/cm^3^ versus 0.17 ± 0.03 mL/cm^3^ in the erlotinib group) (see Table 1 for the tariquidar group and Table 2 for the erlotinib groups).

**Table 1:** Outcome Parameters for [^11^C]Erlotinib Distribution to the Retina and Whole Brain Gray Matter for the Baseline Scan and the Scan during P-gp Inhibition with Tariquidar^a^

region of interest	group	*V*_T_ (mL/cm^3^)	AUC_tissue_ (SUV × min)	AUC_plasma_ (SUV × min)	AUC_tissue_/AUC_plasma_
retina	baseline (*n* = 5)	0.89 ± 0.18 (6)	34.0 ± 3.9	56.1 ± 12.1	0.62 ± 0.14
	tariquidar (*n* = 5)	0.66 ± 0.11* (2)	37.8 ± 9.4	76.4 ± 22.3*	0.51 ± 0.09*
whole brain gray matter	baseline (*n* = 5)	0.18 ± 0.02 (1)	10.4 ± 2.1	56.1 ± 12.1	0.19 ± 0.02
	tariquidar (*n* = 5)	0.22 ± 0.04 (1)	14.8 ± 3.7	76.4 ± 22.3*	0.20 ± 0.03

a Values are reported as the arithmetic mean ± standard deviation. The values in parentheses represent the precision of the parameter estimates (expressed as their mean standard error in percent). *V*_T_ (mL/cm^3^), total volume of distribution estimated with Logan graphical analysis; AUC_tissue_, area under the retina/brain time–activity curve; AUC_plasma_, area under the plasma time–activity curve; AUC_tissue_/AUC_plasma_, ratio of the AUC in tissue (retina or brain) to AUC_plasma_. *p < 0.05 for comparison with the baseline scan using a two-sided paired *t*-test.

**Table 2:** Outcome Parameters for [^11^C]Erlotinib Distribution to the Retina and Whole Brain Gray Matter for the Baseline Scan and the Scan during BCRP/P-gp Inhibition with Orally Administered Erlotinib^a^

region of interest	group	*V*_T_ (mL/cm^3^)	AUC_tissue_ (SUV × min)	AUC_plasma_ (SUV × min)	AUC_tissue_/AUC_plasma_
retina	baseline (*n* = 17)	0.69 ± 0.14 (5)	28.5 ± 5.3	59.5 ± 13.1	0.49 ± 0.09
	erlotinib 300 mg (*n* = 7)	0.40 ± 0.10* (2)	27.8 ± 6.6	81.2 ± 16.7*	0.34 ± 0.06*
	erlotinib 650 mg (*n* = 8)	0.39 ± 0.10* (5)	32.0 ± 8.8	100.5 ± 18.8*	0.32 ± 0.07*
	erlotinib 1000 mg (*n* = 2)	0.33 (2); 0.56 (6)	16.8; 36.5	69.6; 87.0	0.24; 0.42
	all doses (*n* = 17)	0.40 ± 0.10* (4)	29.6 ± 8.2	89.9 ± 19.4*	0.33 ± 0.07*
whole brain gray matter	baseline (*n* = 17)	0.17 ± 0.03 (1)	9.9 ± 2.2	59.5 ± 13.1	0.17 ± 0.03
	Erlotinib 300 mg (*n* = 7)	0.18 ± 0.03 (1)	14.3 ± 2.8*	81.2 ± 16.7*	0.18 ± 0.03
	erlotinib 650 mg (*n* = 8)	0.19 ± 0.04* (1)	18.3 ± 2.7*	100.5 ± 18.8*	0.19 ± 0.03*
	erlotinib 1000 mg (*n* = 2)	0.26 (1); 0.26 (1)	17.4; 22.8	69.6; 87.0	0.25; 0.26
	all doses (*n* = 17)	0.198 ± 0.04* (1)	16.9 ± 3.6 *	89.9 ± 19.4*	0.19 ± 0.04*

a Values are reported as arithmetic mean ± standard deviation except for the 1000 mg erlotinib dose group, for which individual values are given. The value in parentheses represents the precision of the parameter estimates (expressed as their mean standard error in percent). *V*_T_ (mL/cm^3^), total volume of distribution estimated with Logan graphical analysis; AUC_tissue_, area under the retina/brain time–activity curve; AUC_plasma_, area under the plasma time–activity curve; AUC_tissue_/AUC_plasma_, ratio of the AUC in tissue (retina or brain) to AUC_plasma_. *p < 0.05 for comparison with baseline scan using the mixed-effects analysis with the Geisser–Greenhouse correction and the Sidak’s multiple comparisons test.

Following P-gp inhibition with tariquidar, a statistically significant decrease in *V*_T_ and AUCR of [^11^C]erlotinib was observed for the retina (*V*_T_: −25 ± 8%, *p* ≤ 0.05; AUCR: −18 ± 8%, *p* ≤ 0.05). This contrasted with the brain, for which no significant changes in *V*_T_ or AUCR of [^11^C]erlotinib were observed after the infusion of tariquidar (Table 1).

**Figure 3:**
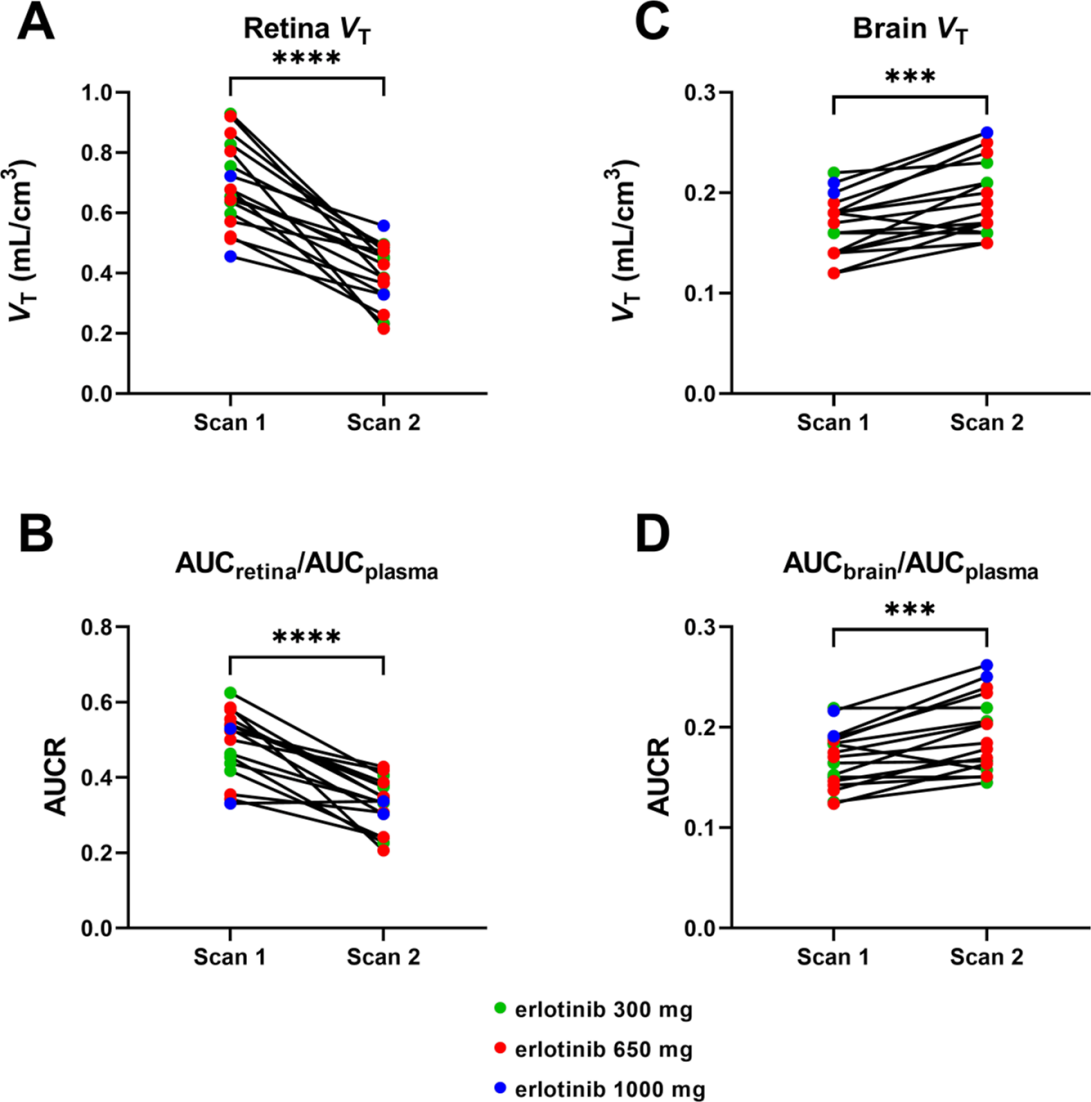
Outcome parameters for [^11^C]erlotinib tissue distribution (total volume of distribution, *V*_T_, and ratio of AUC in tissue to AUC in plasma, AUCR) for the retina (A,B) and whole brain gray matter (C,D) for the baseline scan (scan 1) and the scan after BCRP and P-gp inhibition (scan 2) with erlotinib at doses of 300 mg (green circles), 650 mg (red circles), or 1000 mg (blue circles). ***p ≤ 0.001, ****p ≤ 0.0001; paired two-sided *t*-test.

Also, after administration of different oral erlotinib doses, a decrease in retinal distribution of [^11^C]erlotinib as compared with the baseline scan was observed (Figure 3, Table 2). This decrease was statistically significant for the 300 mg dose group (*V*_T_: −44 ± 12%, *p* ≤ 0.05 and AUCR: −34 ± 9%, *p* ≤ 0.05), for the 650 mg dose group (*V*_T_: −42 ± 19%, *p* ≤ 0.05 and AUCR: −31 ± 21%, *p* ≤ 0.05), and for all combined erlotinib dose groups (*V*_T_: −41 ± 16%, *p* ≤ 0.001 and AUCR: −31 ± 15%, *p* ≤ 0.001). It was not possible to perform statistical analysis for the 1000 mg dose group due to the small sample size.

Similar to tariquidar administration, the effect of oral erlotinib administration on the distribution of [^11^C]erlotinib to the retina was different from that in the brain. For the brain, no statistically significant changes in *V*_T_ and AUCR in the 300 mg erlotinib dose group were observed, while a statistically significant increase in *V*_T_ and AUCR was observed for the 650 mg dose group and all combined erlotinib doses (for all combined erlotinib doses: *V*_T_: +20 ± 16%, *p* ≤ 0.001; AUCR: +15 ± 14%, *p* ≤ 0.001) (Table 2 and Figure 3). In contrast to the brain for which the percentage change in AUCR or *V*_T_ in scan 2 was positively correlated with erlotinib plasma exposure until the end of the imaging session (AUC_plasma,0–4 h_, *r* = 0.828, *p* ≤ 0.001, and *r* = 0.862, *p* ≤ 0.001, respectively), no correlation could be found for the retina between the changes in AUCR or *V*_T_ and AUC_plasma,0–4 h_ (data not shown).

### Effect of P-gp/BCRP Inhibition on [^3^H]Erlotinib Distribution to the Mouse Brain and Retina

**Figure 4:**
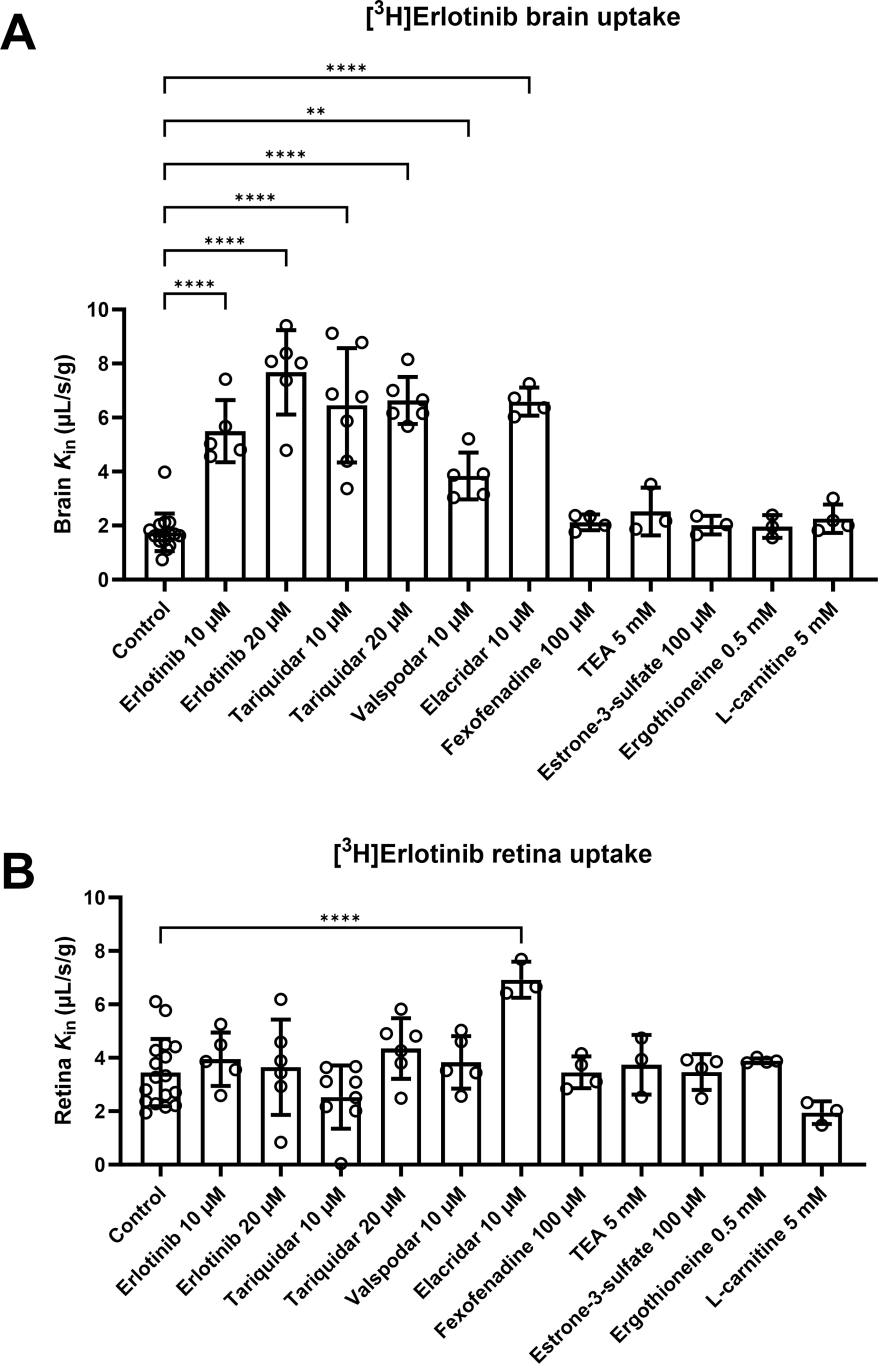
Effect of ABC transporter inhibitors (10 or 20 μM erlotinib, 10 or 20 μM tariquidar, 10 μM valspodar, 10 μM elacridar) and SLC transporter inhibitors (100 μM fexofenadine, 5 mM TEA, 100 μM estrone-3-sulfate, 0.5 mM ergothioneine, 5 mM l-carnitine) on [^3^H]erlotinib transport at the mouse BBB (A) and BRB (B), measured by in situ carotid perfusion. Data represent the mean ± SD (*n* = 3–17 mice per group). **p ≤ 0.01; ***p ≤ 0.001; ****p ≤ 0.0001; one-way ANOVA with a Dunnett’s multiple comparison test for comparisons with the control group (without inhibitor).

At the mouse BBB, the initial transport rate of [^3^H]erlotinib (*K*_in_) at baseline (1.75 ± 0.70 μL/s/g) was significantly increased after P-gp and/or BCRP inhibition with 10 μM erlotinib (+215%), 20 μM erlotinib (+340%), 10 μM tariquidar (+270%), 20 μM tariquidar (+280%), 10 μM valspodar (+120%), and 10 μM elacridar (+277%) (Figure 4A). At the mouse BRB, *K*_in_ of [^3^H]erlotinib was approximately 2 times higher than at the BBB and was only significantly increased after P-gp/BCRP inhibition with elacridar but not after co-perfusion with erlotinib, tariquidar, or valspodar (Figure 4B). In elacridar-co-perfused animals, the magnitude of the increase in [^3^H]erlotinib distribution to the retina (+114%) was markedly lower than for the brain (+277%).

### Effect of SLC Transporter Inhibition on [^3^H]Erlotinib Distribution to the Mouse Brain and Retina

Co-perfusion with prototypical SLC transporter inhibitors was performed in the in situ carotid perfusion experiments in order to investigate a possible involvement of a range of organic cation transporters (OCTs), novel OCTs (OCTNs), and organic anion-transporting polypeptides (OATPs) on the cerebral and retinal distribution of [^3^H]erlotinib. None of the inhibitors (fexofenadine, TEA, estrone-3-sulfate, ergothioneine, and l-carnitine) led to significant changes in brain or retina [^3^H]erlotinib transport (Figure 4). Moreover, co-infusion of two different doses of unlabeled erlotinib did not decrease the *K*_in_ of [^3^H]erlotinib in the retina. Altogether, this suggests the lack of involvement of the investigated SLC transporters in the cerebral and retinal distribution of [^3^H]erlotinib in mice.

## Discussion

In this study, PET imaging was used to assess the functional impact of transporters on controlling the distribution of the dual P-gp/BCRP substrate [^11^C]erlotinib to the human retina. This study complements two previous studies from our group, in which we used PET to assess the impact of P-gp and/or BCRP on the retinal distribution of two other radiolabeled transporter substrates in humans, i.e., the P-gp substrate (*R*)-[^11^C]verapamil and the dual P-gp/BCRP substrate [^11^C]tariquidar.^17,18^ As opposed to these two previous PET studies, in which P-gp inhibition resulted in qualitatively similar effects at the BRB and BBB—i.e., increases in the tissue distribution of the radiolabeled transporter substrates^17,18^—our present study revealed dissimilar effects of transporter inhibition with respect to the retinal and cerebral distribution of [^11^C]erlotinib.

Our previous studies have shown that [^11^C]erlotinib is hardly metabolized over the duration of the PET scan in both humans and mice.^22,27^ The majority (>90%) of radioactivity in human and mouse plasma and mouse brain was composed of unmetabolized [^11^C]erlotinib, and we therefore assumed in the present study that the PET signal in human retina and brain tissue consisted only of [^11^C]erlotinib. Two approaches for ABC transporter inhibition and saturation were used to modulate [^11^C]erlotinib tissue distribution. As a first approach, we employed a previously described i.v. co-infusion protocol of the well-known P-gp inhibitor tariquidar.^28^ At in vivo achievable human plasma concentrations, tariquidar only inhibits P-gp and not BCRP at the BBB.^9^ This tariquidar administration protocol was shown to lead to an approximately 4-fold increase in the brain distribution of the P-gp substrate (*R*)-[^11^C]verapamil.^28^ As shown for the brain, the retinal distribution of (*R*)-[^11^C]verapamil was also increased following tariquidar co-infusion, albeit to a lower extent than for the brain (i.e., a 1.4-fold increase in retinal *V*_T_).^18^ This demonstrated that P-gp is functionally active at the human BRB but that it is likely less abundant at the BRB than at the BBB. In contrast to (*R*)-[^11^C]verapamil, this tariquidar administration protocol led to only negligible increases in the brain distribution of the dual P-gp/BCRP substrates [^11^C]tariquidar, [^11^C]elacridar, and [^11^C]erlotinib.^9,22^ This is in agreement with functional redundancy between P-gp and BCRP at the BBB, which both need to be simultaneously inhibited to substantially increase the brain distribution of dual P-gp/BCRP substrates.^7,8^ However, in heterozygous carriers of the reduced-function *ABCG2* single-nucleotide polymorphism (SNP) c.421C > A, the brain distribution of [^11^C]tariquidar was significantly increased following co-infusion of tariquidar, presumably due to decreased BCRP activity at the BBB.^9^ A similar effect was observed for the retina, in which the distribution of [^11^C]tariquidar was only increased in SNP carriers (c.421CA) and not in noncarriers (c.421CC) following tariquidar co-infusion.^17^ This provided first in vivo evidence that both P-gp and BCRP are functionally active at the human BRB. As a second inhibition approach, we administered single ascending oral doses of erlotinib, up to 6 times higher dose (i.e., 1000 mg) than the clinically employed dose of 150 mg.^22^ In vitro data showed that erlotinib is a potent inhibitor of BCRP, which additionally, but less potently, inhibits P-gp.^24^ In a previous study, we found that the brain distribution of a microdose of [^11^C]erlotinib was significantly increased in humans following oral high-dose erlotinib administration.^22^ This can be explained by BCRP being more abundant at the human BBB than P-gp,^29^ leading to a greater effect of BCRP inhibition/saturation (by erlotinib) than P-gp inhibition (by tariquidar) on the brain distribution of dual P-gp/BCRP substrates. This is also supported by data obtained in nonhuman primates in whom either tariquidar infusion or high-dose erlotinib infusion led to only negligible increases in [^11^C]erlotinib brain distribution, while simultaneous co-infusion of both inhibitors resulted in a substantial 4-fold increase in [^11^C]erlotinib brain distribution.^30^ In the present study, we extended the analysis of our human [^11^C]erlotinib PET data set^22^ to compare the effect of tariquidar co-infusion or oral pretreatment with erlotinib on [^11^C]erlotinib distribution to the retina and the brain.

Baseline retinal distribution of [^11^C]erlotinib (*V*_T_, AUCR) was about 3–4 times higher than its cerebral distribution. This finding is similar to the results obtained with [^11^C]tariquidar, whose baseline distribution (*V*_T_) was 4–5 times higher in the human retina than the human brain.^17^ This is in good agreement with animal data suggesting a higher abundance and activity of P-gp and BCRP at the BBB than at the BRB.^11,13,14,31^ Interestingly, quantitative proteomics data from pigs indicated that while the overall abundance of P-gp and BCRP is lower at the porcine iBRB than at the porcine BBB, BCRP is at both barriers approximately 3-fold more abundant than P-gp.^14^ In contrast, *V*_T_ of (*R*)-[^11^C]verapamil was similar for the retina and the brain in humans,^18^ which may be related to the differential expression of a verapamil uptake transporter at the BBB and the BRB.^11^

In contrast to the brain, administration of both tariquidar and erlotinib led to a significantly decreased retinal distribution of [^11^C]erlotinib. We hypothesized that the observed discrepancy between the effects of tariquidar/erlotinib administration on the retinal and cerebral distribution of [^11^C]erlotinib may be caused by the involvement of an uptake transporter at the BRB, which mediates plasma-to-retina transfer of [^11^C]erlotinib and which is also inhibited or saturated by tariquidar or erlotinib. Thus, inhibition or saturation of a retinal uptake transporter may attenuate the increase in retinal distribution of [^11^C]erlotinib following ABC efflux transporter inhibition, resulting in a net decrease in retinal distribution of [^11^C]erlotinib. We previously found that erlotinib is at low concentrations transported by human OATP2B1 (encoded by the *SLCO2B1* gene) and obtained evidence that in vivo liver uptake of [^11^C]erlotinib in humans is at least partly mediated by OATP2B1.^32^ Moreover, erlotinib is a potent OATP2B1 inhibitor.^33,34^ The expression of OATP2B1 is not confined to the liver, it is among other tissues also expressed in human brain capillary endothelial cells and in the retina, i.e., in neural cells within the inner nuclear and inner plexiform layers and in the RPE.^35,36^ Next to OATP2B1, OATP1A2 (encoded by the *SLCO1A2* gene) is also abundantly expressed in the human retina.^35^ There is further evidence that erlotinib is a substrate or inhibitor of OCTs or OCTNs,^20,34,37^ which are also expressed at the BRB.^4^ Finally, in vitro data indicate that tariquidar is a substrate of OCTN1 (encoded by the *SLC22A4* gene)^38^ and an inhibitor of OATP1A2.^39^

To elucidate a possible functional interplay between ABC efflux and SLC uptake transporters in the retinal and cerebral distribution of erlotinib, we performed in situ carotid perfusion experiments in mice. These experiments revealed a comparable discrepancy between the retinal and cerebral distribution of [^3^H]erlotinib in mice as in humans (Figure 4). While co-perfusion with erlotinib, tariquidar, valspodar, and elacridar led to significant increases in brain transport (*K*_in_) of [^3^H]erlotinib, the retina *K*_in_ was only significantly increased with elacridar co-perfusion. The perfusion fluid employed in the in situ carotid perfusion experiments does not contain protein, and the employed inhibitor concentrations were several times higher than the respective unbound plasma concentrations in humans (approximately 0.4 μM for erlotinib and 0.03 μM for tariquidar).^9,22^ At the concentrations employed in the in situ carotid perfusion experiments, erlotinib, tariquidar, and elacridar are expected to inhibit both P-gp and BCRP at the mouse BRB and BBB, while valspodar selectively inhibits P-gp.^40^ The increased retinal and cerebral uptake of [^3^H]erlotinib in elacridar-co-perfused animals confirmed that P-gp and BCRP limited the distribution of erlotinib to the mouse retina and brain, with a greater transporter impact in the brain. The markedly greater effect of co-perfusion with elacridar (dual P-gp/BCRP inhibitor) than valspodar (P-gp inhibitor) on retinal uptake of [^3^H]erlotinib (Figure 4B) may suggest that BCRP is at the mouse BRB more abundant than P-gp. However, the lack of an effect of tariquidar or erlotinib co-perfusion on retinal uptake of [^3^H]erlotinib as opposed to its cerebral uptake was in line with our hypothesis of the presence of a carrier-mediated uptake mechanism for erlotinib at the BRB, which may have concealed the effects of efflux transporter inhibition. We therefore tested the effect of co-perfusion with prototypical inhibitors/substrates of SLC transporters on the retinal and cerebral distribution of [^3^H]erlotinib. We employed fexofenadine^41^ and estrone-3-sulfate^42^ as OATP substrates/inhibitors, TEA as a substrate/inhibitor of OCTs (OCT1-3, *SLC22A1-3*),^43^ ergothioneine as a substrate/inhibitor of OCTN1,^44^ and l-carnitine as a substrate/inhibitor of OCTN2 (*SLC22A5*).^45^ However, none of these inhibitors had an effect on the retinal and cerebral distributions of [^3^H]erlotinib, which argued against an involvement of these transporters.

A commonly used parameter to assess the sum of transporter effects on the extent of unbound drug passage across biological barriers is the unbound tissue-to-plasma concentration ratio (*K*_p,uu_).^46^ The PET outcome parameter *V*_T_, on the other hand, equals the tissue-to-plasma concentration ratio of total (i.e., bound and unbound) radioactivity. Changes in *V*_T_ may therefore be due to either changes in transporter activity or changes in tissue binding. An alternative hypothesis for the observed differences in retinal *V*_T_ between the two PET scans is therefore that erlotinib binds to some structure in the retina, which is absent in the brain, such as melanin. This is supported by the observation that [^11^C]erlotinib kinetics in the retina appeared to be less reversible than those in the brain (Figure 2). The RPE forming the oBRB together with choroid tissues contains most of the eumelanin, which is the ocular melanin type mainly involved in drug binding.^5,47^ A competition for melanin binding between [^11^C]erlotinib and tariquidar or unlabeled erlotinib may thus have masked the effects of efflux transporter inhibition in the human retina and may explain the decrease in [^11^C]erlotinib distribution to the retina in the case of concomitant administration of tariquidar or erlotinib. Radiolabeled tariquidar but also radiolabeled gefitinib, a first-generation EGFR-tyrosine kinase inhibitor like erlotinib, were shown to accumulate in melanin-rich tissues in animals, particularly in the eye.^48,49^ Despite the lack of specific data in the literature on the binding capacity of erlotinib to melanin, its structural and physicochemical similarities to gefitinib (substituted quinazolines, p*K*_a_ 5.4 for both drugs)^34^ suggest that erlotinib could also bind to melanin in the human retina. It should be noted, however, that any possible melanin binding of erlotinib would not play a role in the in situ carotid perfusion experiments in which albino mice were used.

Ocular toxicity of molecularly targeted anticancer agents is not necessarily limited to retinotoxicity.^2^ Erlotinib-induced eye toxicity was hypothesized to be linked to inhibition of EGFR, which is localized in the cornea and conjunctival epithelial cells.^50^ Systemic application of erlotinib was associated with toxic manifestations on the ocular surface, such as conjunctivitis, keratoconjunctivitis sicca, blepharitis, corneal perforation or ulceration, and trichomegaly^1,2,51^ but not with retinotoxicity.^19,52^ However, retinal adverse events (e.g., retinal detachment, retinal vascular occlusion, optic neuropathy) were observed with other molecularly targeted anticancer drugs, such as mitogen-activated protein kinase (MEK) inhibitors, which interfere with the MAPK pathway thought to be involved in retinal homeostasis,^2,52,53^ but also with fibroblast growth factor receptor (FGFR) inhibitors and the BCR-ABL tyrosine kinase inhibitor imatinib.^52^ Since most of these drugs are substrates of P-gp and BCRP,^54^ transporter activity at the BRB may play a role in their retinal distribution and retinal toxicities.

## Conclusions

Our study highlights the great potential of PET imaging to noninvasively measure drug distribution to the human retina and to assess the impact of membrane transporters expressed in blood-ocular barriers. In situ carotid perfusion experiments in mice suggested that both P-gp and BCRP limit the distribution of erlotinib to the mouse retina and brain, but that the impact of these transporters was greater at the BBB than at the BRB. However, a dissimilar effect of tariquidar or erlotinib administration on the retinal and cerebral distribution of [^11^C]erlotinib was observed in humans, which may be related to the presence of an unidentified erlotinib uptake transporter at the BRB, which is not present at the BBB or concealed by ABC transporter efflux. Given that most known molecularly targeted anticancer drugs are substrates of ABC and SLC transporters and that some of these drugs display retinotoxicity, it appears possible that membrane transporter activity at the BRB may play a role in these ocular adverse effects.
